# Empirical Comparison of Sources of Variation for FMRI Connectivity Analysis

**DOI:** 10.1371/journal.pone.0003708

**Published:** 2008-11-12

**Authors:** Baxter P. Rogers, John C. Gore

**Affiliations:** 1 Vanderbilt University Institute of Imaging Science, Vanderbilt University, Nashville, Tennessee, United States of America; 2 Department of Radiology and Radiological Sciences, Vanderbilt University, Nashville, Tennessee, United States of America; 3 Department of Molecular Physiology and Biophysics, Vanderbilt University, Nashville, Tennessee, United States of America; 4 Department of Biomedical Engineering, Vanderbilt University, Nashville, Tennessee, United States of America; 5 Department of Physics, Vanderbilt University, Nashville, Tennessee, United States of America; Albert Einstein College of Medicine, United States of America

## Abstract

**Background:**

In neuroimaging, connectivity refers to the correlations between signals in different brain regions. Although fMRI measures of connectivity have been widely explored, the methods used have varied. This complicates the interpretation of existing literature in cases when different techniques have been used with fMRI data to measure the single concept of “connectivity.” Additionally the optimum choice of method for future analyses is often unclear.

**Methodology/Principal Findings:**

In this study, measures of functional and effective connectivity in the motor system were calculated based on three sources of variation: inter-subject variation in task activation level; within-subject variation in task-related responses; and within-subject residual variation after removal of task effects. Two task conditions were compared. The methods yielded different inter-regional correlation coefficients. However, all three approaches produced similar results, qualitatively and sometimes quantitatively, for condition differences in connectivity.

**Conclusions/Significance:**

While these results are specific to the motor regions studied, they do suggest that within-subject and across-subject results may be usefully compared. Also, the presence of task-specific correlations in residual time series supports arguments that residuals may not substitute for resting-state data, but rather may reflect the same underlying variations present during steady-state performance.

## Introduction

Measuring connectivity from functional MRI data holds promise as a non-invasive approach to understanding the function and architecture of the brain and how neural circuits change in psychiatric disorders. Studies of connectivity to date have implicated connectivity changes in such conditions as schizophrenia [1], depression [2], autism [3], and reading disorders [4], [5]. In neuroimaging, functional connectivity is generally defined in terms of the correlations between signals from different anatomical regions; effective connectivity studies attempt to make inferences about influences of brain regions on each other [6]. However, there are a wide diversity of measures that have been labeled as “connectivity;” this makes interpretation of the existing literature problematic, and leads to the question of which techniques might be best for future work.

Several different techniques for measuring connectivity have been reported in the neuroimaging literature: principal components analysis, seed-voxel correlation maps, and structural equation modeling, for instance. The majority of these techniques are based on correlations or covariances calculated between measured signals in different voxels or regions of interest; see [7] for a review. However, the precise source of signal variation that is used can vary widely and results of any study will depend on the specific choices made [8]. For fMRI data, the variation that drives inter-regional connectivity measures can be inter-subject or within subject; within-subject variation can be across time, experimental condition, block, or trial [9]. In practice, multiple sources of variation are often present in a data set. There is no certainty that different choices in this regard will yield similar results, so connectivity measures in general must be interpreted in light of the specific methods used to produce them. Even considering only time series data within subject, inter-regional correlations depend on which components of variation are retained. For instance, it has been demonstrated in simulated data and in a visual fMRI experiment that correlations varied depending on whether task-related variance, block-to-block variance, and residual variance were removed from the time series [10]. Differences between resting-state data and the residuals from block and event-related stimulation experiments in terms of inter-regional correlations have been found as well [11]. A specific example of the difficulty that arises in interpreting the existing literature is shown in Table 1; two studies of schizophrenic patients [12], [13] reported apparently conflicting results but there is no way to determine whether the conflict is intrinsic to the data or is produced by the difference in methodology.

**Table 1:** Conflicting Connectivity Results.

Study	Underlying variance	Connectivity model	Left cer. to PFC	Right cer. to PFC	Left cer. to thal.
Stephan 2001 [12]	Task, scan	Functional (seed voxel maps)	Higher in patients	Lower in patients	Lower in patients
Schlosser 2003 [13]	Subject, task, scan	Effective (structural equation model)	Lower in patients	Lower in patients	No difference

These results from two studies of schizophrenic patients were chosen to exemplify two methodological approaches. The table reports connectivity between cerebellum, prefrontal cortex, and thalamus in olanzapine-treated patients with respect to non-schizophrenic control subjects. Synthesis of the two results is confounded by differences in methodology.

In this study, we calculated three different measures of connectivity from the same block-design fMRI data set to see whether they gave similar conclusions. Two within-subject methods were used, one considering task-related variance and the other considering residual variance after the removal of task effects. The third method considered inter-subject variance in activation measures. For each method we calculated functional connectivity in each of two experimental conditions (raw correlation scores); the condition differences in functional connectivity; and the condition differences in effective connectivity using a regression model.

## Methods

Functional MRI data from a finger-tapping study [14] were used. Written informed consent was obtained from all subjects, under a protocol approved by the University of Wisconsin-Madison institutional review board. Ten subjects performed right hand movement and left hand movement in separate sessions, with 7 14-second blocks of movement paced by auditory tones interspersed with 14-second blocks of no motion. Scan repetition time was 1.75 seconds. Regions of interest in right and left supplementary motor area (SMA) and right and left sensorimotor cortex (SMC) were defined based on the anatomical criteria described in the original work.

### Region of interest data

Data analysis was performed using the AFNI software [15], [16] and Matlab (The MathWorks, Inc., Natick MA). Values at each voxel were shifted in time to account for differences in slice acquisition time, then the functional volumes for each subject were coregistered to correct for head motion. These data were spatially smoothed with a Gaussian filter of 5 mm FWHM. Voxel time series for each session were then pre-processed to remove movement-related variations of no interest and very low-frequency drifts by regressing on the previously estimated motion parameters and 0th–4th order Legendre polynomials as predictors. The residual from this regression was used for further analysis after scaling each voxel time series by its estimated mean to obtain approximate percent change units. Stimulus-related effects were identified by fitting a general linear model whose predictors contained delta functions located at a constant offset from the task block onsets; in other words, a standard linear deconvolution approach to estimating the hemodynamic impulse response function without assumptions on its shape (see [17] for a description and evaluation of the method). The estimated impulse response was a vector with 16 elements representing scans after block onset. An activation measure was calculated from the impulse response as the difference between the average value in the movement block and the average value within the rest block, after discarding the first three time points of each to allow for transition effects. The activation measure was implemented as a contrast in the voxel-wise linear model, permitting statistical inference. To avoid including voxels with minimal task-related signal that were nonetheless within the anatomically defined ROIs, we reduced ROIs to include only the 15 voxels with the highest significance of the activation measure across both sessions.

Region of interest data were averaged over the selected voxels. The **Task method** used the estimated impulse response functions, which contain variations in the hemodynamic signal over time, as reproduced in all blocks. The **Residual method** used the residuals from the second linear model, which had all reproduced stimulus-related effects partialed out but may still have contained block-to-block variations. The **Subject method** used the single activation measure. Therefore for each ROI the Task and Residual data varied over time within subject; the Subject data varied across subjects only.

### Functional connectivity

For each of the three sets of ROI data, the correlation coefficient *R* between the signals from each pair of ROIs was calculated. The correlations were converted to Z-scores using the Fisher transformation 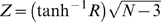 to produce approximately normal random variables with variance 1 [18]. The degrees of freedom N enter in this calculation; for the Subject data, N = 10 subjects. For the Task data, N = 16, the number of estimated points in the impulse response. For the Residual data, N was the effective degrees of freedom accounting for the temporal dependence in the data. Autocorrelation was present in the residuals of one or more ROIs in 9 of 10 subjects (p<0.01, Durbin-Watson test). Hence, for each ROI pair N was calculated as the number of volumes (112) multiplied by the factor (1−*ρ* ^2^)/(1+*ρ* ^2^) where *ρ* was the estimated first order autocorrelation. This correction procedure gives a reasonable approximation for the effective degrees of freedom in fMRI time series [19]. For Task and Residual methods, the correlation measures for each subject were averaged to obtain a single measure for the group for comparison with the Subject method. These calculations resulted in a single measure per method, per subject of correlation during right hand tapping (*Z_R_*) and during left hand tapping (*Z_L_*). To compare connectivity during the two conditions, the value *Z_R_*−*Z_L_* was used, itself an approximately normal variable representing the difference of interest. To compare methods directly on this metric, the measures (*Z_R_*−*Z_L_*)_Residual_−(*Z_R_*−*Z_L_*)_Task_ and so on were used to determine whether different methods produced significantly different measurements of the condition difference *Z_R_*−*Z_L_*.

### Effective connectivity

**Figure 1:**
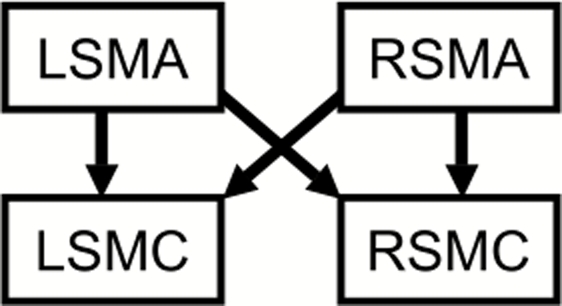
Effective Connectivity Regression Model. This model was fit to the region-of-interest data for each of the three methods to obtain estimates of effective connectivity. The model reflects the inter- and intra-hemispheric influences of interest in the original experiment. L = Left; R = Right; SMA = Supplementary Motor Area; SMC = primary SensoriMotor Cortex.

The 4-parameter regression model of Figure 1 was fit to the ROI data for each method, using ordinary least squares. For the Task and Residual methods, path coefficients were estimated within each subject directly from the time series data, and the path coefficient means over all subjects were calculated, similarly to the method of the original study [14]. For the Subject method, the vectors of activation measures were scaled to mean 0, standard deviation 1, and a single coefficient was estimated for each path, similarly to a previous study of the angular gyrus in dyslexia [4].

### Bootstrap confidence intervals

A bootstrap procedure was used to estimate confidence intervals (CIs) for the normalized correlation values and path coefficients. 1000 bootstrap samples were created by drawing 10 subjects randomly with replacement from the original sample. The complete analysis above was repeated on each new sample, and 90% percentile confidence intervals were calculated for each measure of interest. The confidence intervals allow a simple statistical interpretation of the graphical results: if a confidence interval does not include zero, the measure in question is non-zero with a p-value of 0.1. The implications of using this lenient threshold depend on what result is being considered and are described in each case below. In particular, use of this threshold actually strengthens the conclusions of the direct comparison between methods, which is of most interest.

## Results

### Raw correlation values

**Figure 2:**
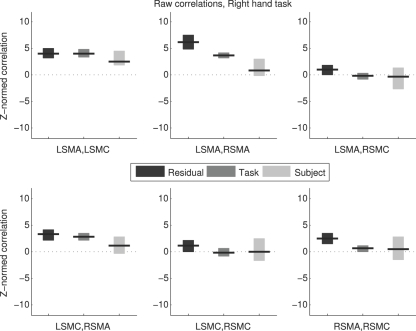
Raw Correlation Values, Right Hand Task. Estimated Z-normalized correlations and 90% bootstrap confidence intervals are shown for each region pair for the right hand movement condition. Each subplot shows results from the three different methods. L = Left; R = Right; SMA = Supplementary Motor Area; SMC = primary SensoriMotor Cortex.

**Figure 3:**
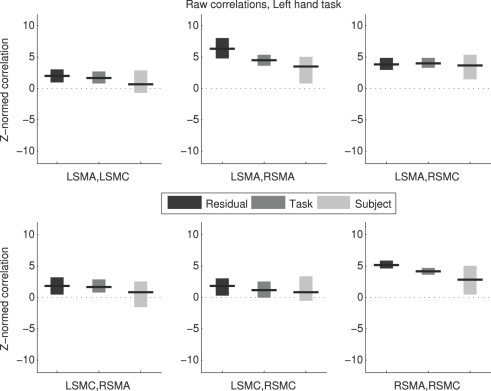
Raw Correlation Values, Left Hand Task. Estimated Z-normalized correlations and 90% bootstrap confidence intervals are shown for each region pair for the left hand movement condition. Each subplot shows results from the three different methods. L = Left; R = Right; SMA = Supplementary Motor Area; SMC = primary SensoriMotor Cortex.

Raw correlation values (*Z_R_* and *Z_L_*) were not very comparable across methods (Figures 2 and 3). Raw correlations measured by the different methods can be compared on two criteria: whether they agree in sign (positive, zero, or negative); and whether they agree in magnitude. All the raw correlation values were either positive (90% CI did not include zero) or not distinguishable from zero (90% CI included zero). However, in some cases, one method found a positive correlation when another method did not; the RSMA/RSMC correlation during right hand movement was an example of this. Agreement in magnitude can be roughly judged based on whether the 90% CIs for two methods overlap; if they do not, the methods certainly do not agree in magnitude at an alpha = 0.05 level. Even if the 90% CIs do overlap, the magnitudes may still be different at alpha = 0.05 (e.g. Payton [20]). Several cases were apparent where CIs of the raw correlation values did not overlap. In summary, Figures 2 and 3 show only partial agreement between methods at best in terms of sign and magnitude of raw correlations.

This was expected regarding the Residual method in particular, as the within-subject values are confounded by respiration, cardiac pulsation, and other signals that manifest differently across subjects. The differences between the Residual method and the Task method add to the findings of [10] as evidence that different time series processing can yield different correlation values within subject.

### Condition differences in correlations

**Figure 4:**
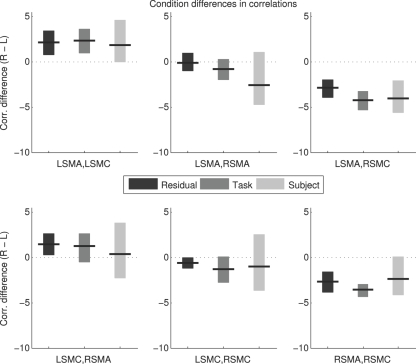
Condition Differences in Correlation Values. Plots show the difference in correlations between the right hand condition and the left hand condition, along with bootstrap 90% confidence intervals. L = Left; R = Right; SMA = Supplementary Motor Area; SMC = primary SensoriMotor Cortex.

The condition difference in the correlation value is the measure of most interest, as it describes the effect of the experimental manipulation on functional connectivity. This is shown for all methods and all region pairs in Figure 4 as the difference between the Z-normalized correlation scores for the right hand and left hand sessions (*Z_R_*−*Z_L_*). All three methods indicate a stronger LSMA/LSMC correlation during right hand movement, and a stronger LSMA/RSMC correlation during left hand movement, based on the 90% confidence intervals (though these comparisons will show some false positives because of the lenient threshold and number of tests). Task and Residual methods indicate a stronger RSMA/RSMC correlation during left hand movement, which is almost mirrored by the Subject method except that the 90% CI in that case barely includes zero. No method indicates a difference in LSMA/RSMA correlation. The Residual method suggests a difference in LSMC/RSMA and LSMC/RSMC correlations that is not reproduced by other methods. In general, there is substantial qualitative agreement between methods. The variability of this measure, however, is slightly higher for the Subject method than for the other two.

**Figure 5:**
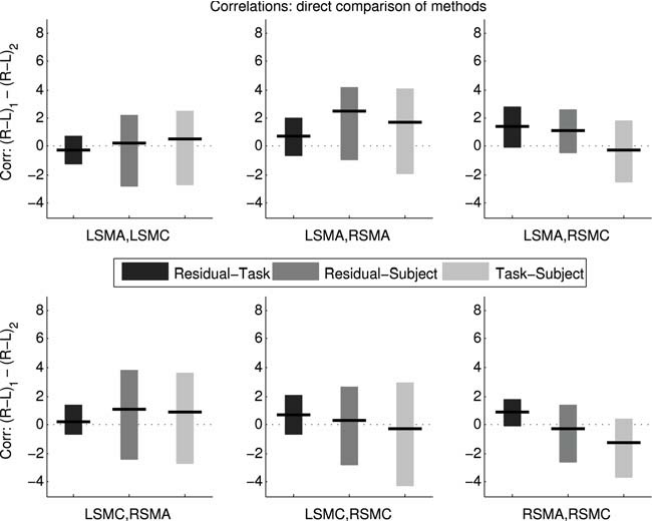
Direct Comparison of Correlation Differences Between Methods. The values of (*Z_R_*−*Z_L_*) from Figure 3 were compared directly between methods. There were no differences in this measure between methods based on the 90% confidence intervals shown. L = Left; R = Right; SMA = Supplementary Motor Area; SMC = primary SensoriMotor Cortex.

Figure 5 shows a direct comparison of the methods. The differences (*Z_R_*−*Z_L_*)_Residual_−(*Z_R_*−*Z_L_*)_Task_ and so on are plotted directly. There is a suggestion of a possible difference between Task/Residual and Task/Subject for the RSMA/RSMC correlation. However, all 90% confidence intervals include zero, indicating no significant differences between methods for any of the correlations. In this case, use of 90% confidence intervals strengthens the conclusion of interest rather than weakening it, because no differences between methods were detected even with such lenient criteria (alpha = 0.1 and multiple comparisons made).

### Condition differences in effective connectivity

**Figure 6:**
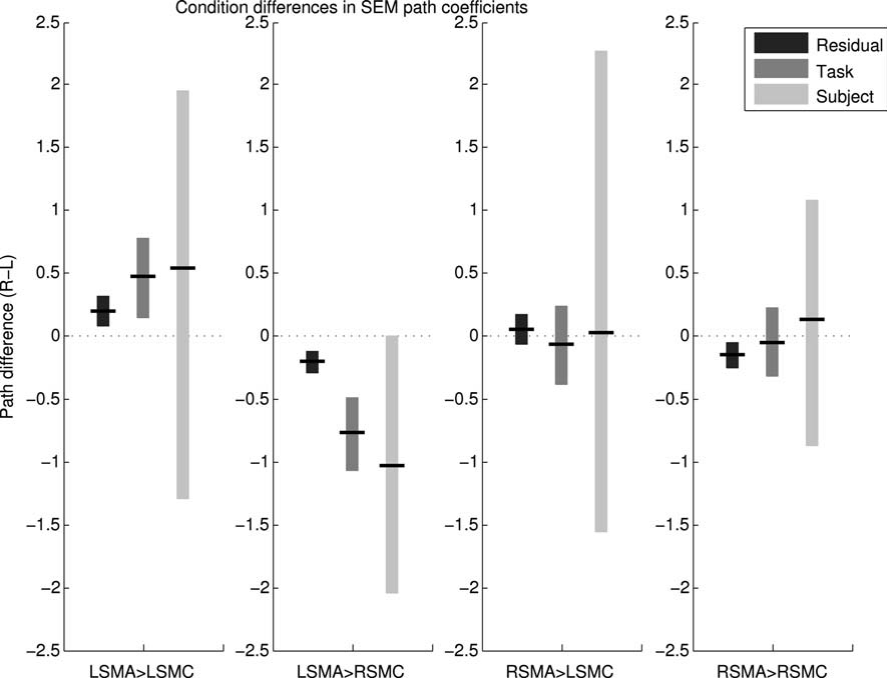
Condition Differences in Path Coefficients. Plots show the difference in coefficients between the right hand condition and the left hand condition, along with bootstrap 90% confidence intervals. All three methods showed greater connectivity from LSMA to RSMC during right hand movement, with no change in RSMA to LSMC connectivity, thereby replicating the key result of the original study. L = Left; R = Right; SMA = Supplementary Motor Area; SMC = primary SensoriMotor Cortex.

Figure 6 shows the condition differences in estimated path coefficients for each method (again, right minus left). All three methods produced two key results: a greater coefficient during left hand movement for the LSMA to RSMC path (p<0.1 for each method; that is, 90% confidence interval does not include zero); and no condition difference for the RSMA to LSMC path (p>0.1). The Task and Residual methods additionally detected a greater LSMA to LSMC coefficient during right hand movement, and the Residual method detected a greater RSMA to RSMC coefficient during left hand movement. In general the magnitudes of the coefficient estimates were not the same for the different methods, so it was only the general conclusions drawn from each regarding effective connectivity that were similar. The variability was substantially higher for the Subject method.

## Discussion

We observed that raw correlation scores were not entirely reproducible across methods. For the Task and Residual methods, this is in accord with [10], who observed that correlation values differed depending on the pre-processing of the data and what components of task-related variability were included. The differences we observed between the Subject method and others broaden this conclusion to include an inter-subject method of calculating correlations. Condition differences in correlation values, however, were better reproduced between methods. Even though the methods are quite different and the underlying variations not the same – orthogonal by construction, in fact, for Task and Residual methods – they all produced the same effect in this data set. Either they all are tied in some way to the same underlying phenomenon, or they are tied to different underlying phenomena that manifest identically in these data. This is a particularly relevant observation for studies that aim to distinguish cognitive networks on the basis of their responses to different tasks or stimuli, though of course it is important to note that these results may be less applicable to other neural systems or cognitive tasks not studied here.

The Residual method produced task-specific changes in connectivity that mirrored the other methods, which suggests that the time series residuals do contain some signals relating to the task context. Therefore they may not make a good substitute for the resting state data used to study characteristics of network interactions that are not task-specific, e.g., [21], [22], [23], in spite of early indications [24]. This has been explored at some length [11], and our results support the observation that correlations in time series residuals did not entirely match the correlations in resting-state data; presumably if the residual correlations matched the resting-state correlations, they would not be affected by the task manipulation. For that reason, time series residuals in connectivity measurement may more appropriately stand in for the steady-state data which has been used to study the motor system [25] and the visual system [26], though this has not been tested directly. However it is worth mentioning in this context that we did not attempt to remove block-to-block variability in the stimulus response, so any that was present was contained in our residual data.

We expect that some of the differences we observed in raw correlation values between the Residual method and the other methods were due to physiological “noise,” generally consisting of cardiac and respiratory signals and head motion artifact and known to confound the measurement of connectivity from time series data. This is of particular relevance to this study, as we did not attempt to remove cardiac, respiratory, or global signals and we used a long TR such that cardiac frequencies would have aliased in an unknown way. Given how the different methods were applied, these signals will have been present in the Residual data but hardly at all in the Task and Subject data (except to the extent they were task-correlated). They will have affected raw correlation values but not condition differences, assuming that they manifested similarly in both sessions for a given subject. Session order was randomized in the original study for precisely this reason.

One difficulty in interpreting the existing functional connectivity literature is the diversity of methodological approaches used [8]. The similarity of results from the three different methods, while limited to the specific tasks studied, do suggest that comparing results from multiple studies may be fruitful in spite of the use of differing methodology.
